# Individual and population pharmacokinetic compartment analysis: a graphic procedure for quantification of predictive performance

**DOI:** 10.3109/21556660.2013.838569

**Published:** 2013-09-03

**Authors:** Staffan Eksborg

**Affiliations:** Department of Women’s and Children’s Health, Childhood Cancer Research Unit, Karolinska Institutet, StockholmSweden

**Keywords:** Pharmacokinetics, Predictive performance, Precision, Accuracy, Statistics

## Abstract

**Objectives:**

Pharmacokinetic studies are important for optimizing of drug dosing, but requires proper validation of the used pharmacokinetic procedures. However, simple and reliable statistical methods suitable for evaluation of the predictive performance of pharmacokinetic analysis are essentially lacking. The aim of the present study was to construct and evaluate a graphic procedure for quantification of predictive performance of individual and population pharmacokinetic compartment analysis.

**Methods:**

Original data from previously published pharmacokinetic compartment analyses after intravenous, oral, and epidural administration, and digitized data, obtained from published scatter plots of observed vs predicted drug concentrations from population pharmacokinetic studies using the NPEM algorithm and NONMEM computer program and Bayesian forecasting procedures, were used for estimating the predictive performance according to the proposed graphical method and by the method of Sheiner and Beal.

**Results:**

The graphical plot proposed in the present paper proved to be a useful tool for evaluation of predictive performance of both individual and population compartment pharmacokinetic analysis.

**Conclusion:**

The proposed method is simple to use and gives valuable information concerning time- and concentration-dependent inaccuracies that might occur in individual and population pharmacokinetic compartment analysis. Predictive performance can be quantified by the fraction of concentration ratios within arbitrarily specified ranges, e.g. within the range 0.8–1.2.

## Introduction

Pharmacokinetic studies are important for optimizing of drug dosing, but requires proper validation of the used pharmacokinetic procedures. Simple and reliable statistical methods suitable for evaluation of the predictive performance of pharmacokinetic analysis, e.g., individual and population compartment pharmacokinetic analysis, are essentially lacking. Poor curve fitting in individual pharmacokinetic compartment analysis can sometimes be recognized from large standard deviations and high correlation of the parameter estimates. Statistical tests for optimization of the number of compartments have been evaluated1–3. Such tests are, however, not suitable for quantification of the predictive performance of pharmacokinetic curve fitting procedures, i.e., for evaluation of the agreement of observed drug concentrations (as determined by bioanalysis) and drug concentrations predicted by the pharmacokinetic curve fitting.

Scatter plots, i.e., plots of observed vs predicted drug concentrations, are often used to illustrate the accuracy of individual and population compartment analysis. Calculated correlation coefficients can be quite misleading, since they assess the degree of association rather than actual closeness of predicted and true values. Rather than to compute a correlation one must realize that the important issue is how well predictions match true (reference) values. Neither a high correlation coefficient, a low *p*-value of the regression line, nor a slope close to unity and a non-significant ordinate at origin are indicators for close agreement between observed and predicted drug concentrations4.

The percentage root mean square prediction error (RMSE%) has been suggested as a measure of precision and the percentage mean prediction error (MPE%) as a measure of bias in pharmacokinetic analysis5. Neither RMSE% nor MPE% are, however, indicators for time- or concentration-dependent inaccuracies.

In this paper the author suggests a plot of the ratio (Drug concentration predicted by pharmacokinetic analysis)/(Drug concentration observed by bioanalysis) vs time (or alternatively vs observed drug concentration) to illustrate the predictive performance of individual and population compartment pharmacokinetic analysis. The predictive performance can be quantified by the fraction of concentration ratios within arbitrarily specified ranges, e.g., within the range 0.8–1.2.

The proposed plot for evaluation of predictive performance of pharmacokinetic analysis is based on the graphical method for evaluation of method-comparison data4. Such plots have also been adopted for evaluation of accuracy of calibration curves6 and for evaluation of the influence of the number of sampling points on the precision and accuracy of the predicted AUC values using a limited sampling strategy7. The applicability of the proposed graphical plot was demonstrated using original data from previously published individual pharmacokinetic compartment analysis after intravenous, oral and epidural administration and digitized data from published scatter plots of observed vs predicted drug concentrations from population pharmacokinetic compartment analysis.

## Materials and methods

### Data collection

Original data from previously published pharmacokinetic compartment analyses after intravenous, oral, and epidural administration8–10 were used for estimating the predictive performance according to the proposed graphical method and by the method of Sheiner and Beal5. Digitized data, obtained from published scatter plots of observed vs predicted drug concentrations from population pharmacokinetic studies using the NPEM algorithm and NONMEM computer program and Bayesian forecasting procedures, were also included in the present study11–13. Figures from original publications were scanned using a Agfa StudioStar scanner (Agfa-Gaevert N.V., Mortsel, Belgium) at 1600 dpi, magnified to ∼11 × 17 cm (landscape orientation) at 600 dpi using Adobe Photoshop CS3 version 10.0.1 (Adobe Systems Inc., San Jose, CA) and printed on a HP Laserjet 1300 printer (Hewlett-Packard Inc., San Diego, CA). The HP 7470 Graphic Plotter equipped with a Digitizing Sight (Part No. 09872-60066, Hewlett-Packard Inc.) with the GraphPad Inplot Software (version 3.0, GraphPad Software Inc., San Diego, CA) was used for digitizing data. The digitizing technique was validated by repeatedly (10 times) digitizing a graph with 20 uniform distributed known data points within the range 5–100% of the length of the axis.

### Statistics

Associations were established by the Spearman rank correlation coefficient. Precision expressed as percentage root mean square prediction error (RMSE%) and bias, expressed as percentage mean prediction error (MPE%), were calculated as outlined by Sheiner and Beal5 using the Microsoft Excel spreadsheet program (Microsoft Excel 97, SR1, Microsoft Corporation, Redmond, WA).

Data were graphed using the GraphPAD Prism program (version 5.03 GraphPad Software Inc.).

## Results

**Figure 1.**
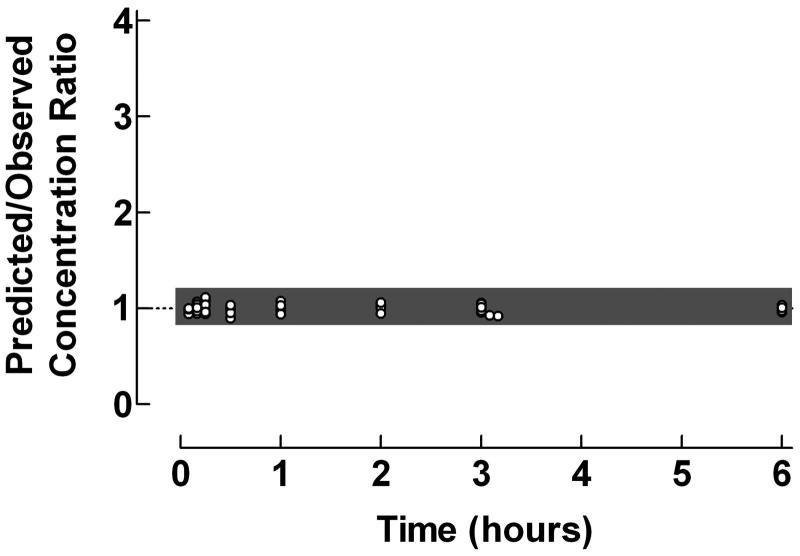
Use of the proposed ratio plot for evaluation of predictive performance of individual pharmacokinetic compartment analysis: intravenous bolus injection of clonidine to children. Shadowed area: Predicted/Observed concentration ratio within the range 0.8–1.2. Data from Lönnqvist and Bergendahl8. The pharmacokinetic data were analyzed by the PC-NONLIN program (version 2.0) using measured serum concentrations as weights in the iterative procedure.

**Figure 2.**
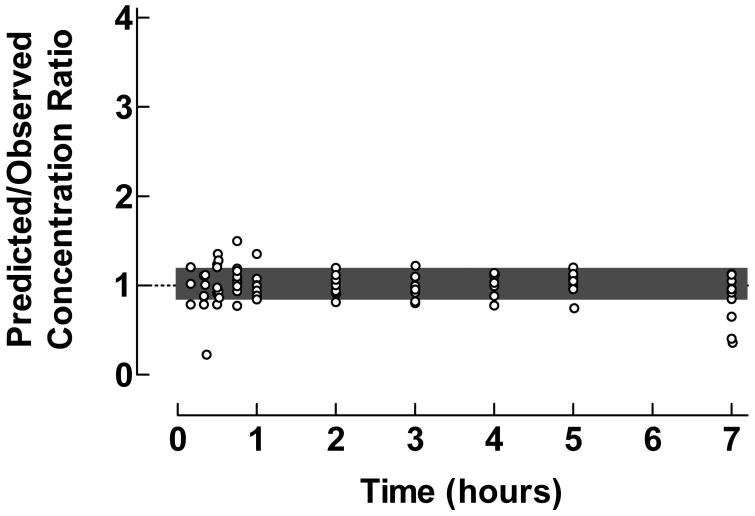
Use of the proposed ratio plot for evaluation of predictive performance of individual pharmacokinetic compartment analysis: Oral administration of the pro-drug valacyclovir to children, with formation to aciclovir. Shadowed area: Predicted/Observed concentration ratio within the range 0.8–1.2. Data from Eksborg *et al*.9. The pharmacokinetic data were analyzed by the PC-NONLIN program (version 2.0) using measured serum concentrations as weights in the iterative procedure.

**Figure 3.**
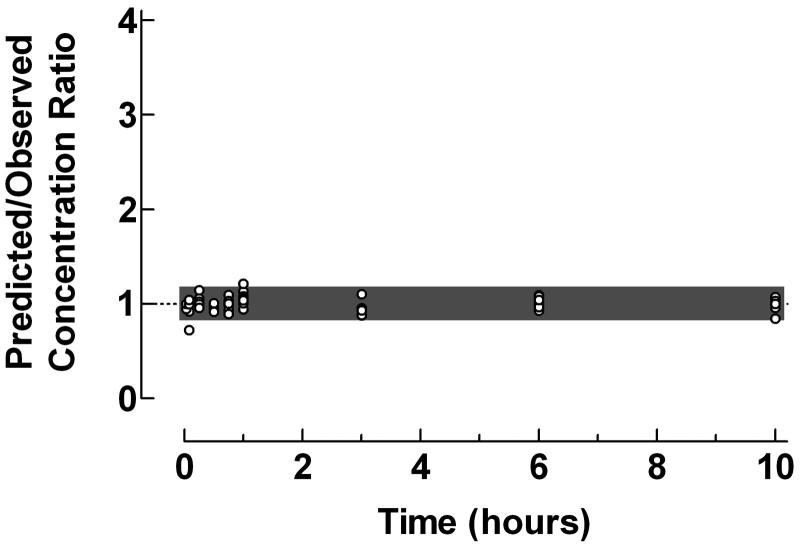
Use of the proposed ratio plot for evaluation of predictive performance of individual pharmacokinetic compartment analysis: Epidural administration of clonidine to pediatric patients. Shadowed area: Predicted/Observed concentration ratio within the range 0.8–1.2. Data from Ivani *et al*.10. The pharmacokinetic data were analyzed by the PC-NONLIN program (version 2.0) using measured serum concentrations as weights in the iterative procedure.

The applicability of the proposed graphical procedure for quantification of predictive performance of individual pharmacokinetic compartment analysis data was demonstrated using original data from previously published pharmacokinetic compartment analyses8–10. Plots of the Predicted/Observed drug concentration ratio vs time, presented in Figures 1–3, illustrate the predictive performance of individual pharmacokinetic compartment analysis using data from intravenous bolus injection of clonidine to children8, oral administration of valacyclovir to children9 and epidural administration of clonidine to children10.

The plasma pharmacokinetics of clonidine after an intravenous bolus injection to children were described using a two-compartment model. The ratio plot in Figure 1 showed an excellent agreement between predicted and observed concentrations with 100% of the concentration ratios within the range 0.8–1.2.

Valacyclovir is an oral pro-drug with a very fast transformation to the active compound aciclovir after absorption. In immunocompromized children with leukopenia and mucositis after chemotherapy, intravenous acyclovir can be substituted by oral valacyclovir9. Pharmacokinetics studies of valacyclovir only comprise quantification of formed aciclovir. From the ratio plot in Figure 2 it is evident that the pharmacokinetic curve fitting during the absorption/transformation phase is somewhat less proper. In general there was a close agreement between predicted and observed aciclovir concentrations during the elimination phase. Some loss of reliability of the curve fitting procedure can also be observed at the last time points, which might impair the evaluation of the terminal half-life times. Still 77.3% of the concentration ratios were within the range 0.8–1.2.

The difficulties in proper compartment analysis of pharmacokinetic data after extravascular administration were previously pointed out14–16. Frequent sampling during the absorption phase and the use of a sophisticated absorption model, i.e., sequential independent zero- and first-order input, enabled successful pharmacokinetic modeling of plasma concentration-time data of clonidine after epidural bolus administration to children10, as confirmed by the ratio plot in Figure 3, with only one deviating data point during the first sampling time, cf. Figure 2. The fraction of concentration ratios within the range 0.8–1.2 was 96.9%.

The applicability of the proposed procedure for quantification of predictive performance of the population pharmacokinetic analysis using three different approaches was, due to lack of original data, demonstrated using digitized data from published scatter plots of observed vs predicted drug concentrations11–13. The precision (RMSE%) and the bias (MPE%) of the digitizing process were 1.085% and −0.347%, respectively, which was considered sufficient for this purpose.

**Figure 4.**
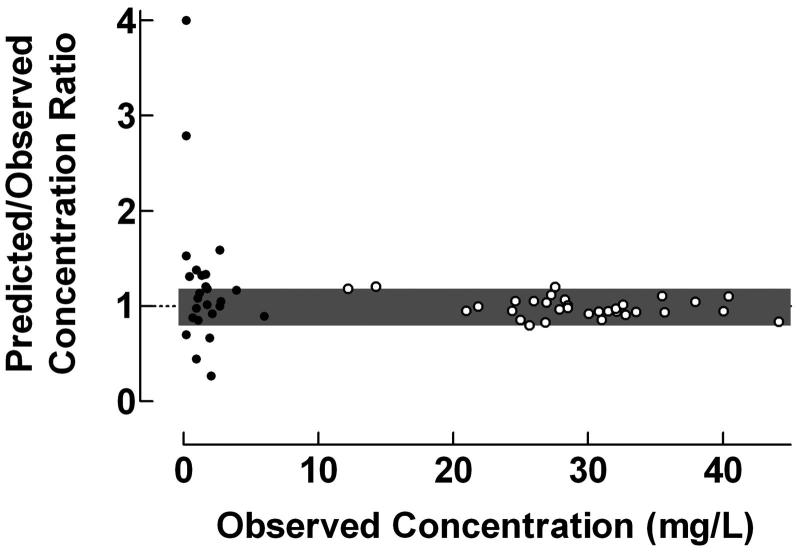
Use of the proposed ratio plot for evaluation of predictive performance: Population pharmacokinetics of amikacin to intensive care patients studied by the NPEM algorithm. Amikacin was administered as 30 min intravenous infusions and the population pharmacokinetics was studied using a one-compartment model. • = trough concentrations; ˆ = peak concentrations. Shadowed area: Predicted/Observed concentration ratio within the range 0.8–1.2. Data from Debord *et al*.11.

The proposed ratio plot was used for evaluation of the predictive performance of the NPEM algorithm, including a one-compartment model, for estimation of trough and peak concentrations of amikacin in intensive care patients after administration as 30 min intravenous infusions11, Figure 4. The ratio plot in Figure 4 revealed a poor prediction of the trough concentrations, but high accuracy and precision in estimated peak concentrations, the fraction of concentration ratios within the range 0.8–1.2 being 48.0% and 93.8% for trough and peak concentrations, respectively. Most likely the imprecision observed in the low concentration range is due to low accuracy of the used analytical methodology6.

**Figure 5.**
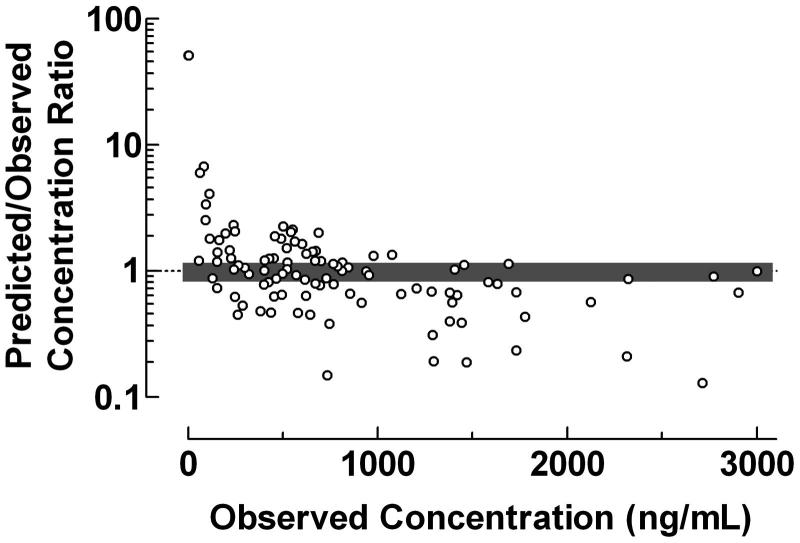
Use of the proposed ratio plot for evaluation of the predictive performance: Population pharmacokinetics of midazolam using the NONMEM computer program. Midazolam was administered as intravenous infusions and bolus injections to neonates. The population pharmacokinetics were studied using a two-compartment model. Shadowed area: Predicted/Observed concentration ratio within the range 0.8–1.2. Data from Burtin *et al*.12.

Data from the validation of the final model using the test data set from a population pharmacokinetic study of midazolam with a two-compartment model using the NONMEM computer program12 after administration of midazolam as intravenous infusions and bolus injections to neonates were used for construction of the ratio plot in Figure 5. The large scatter of the data indicates a poor performance of the used population pharmacokinetic procedure within the entire concentration range. Only 29.4% of concentration ratios were within the range 0.8–1.2. The Predicted/Observed concentration ratio showed a tendency to decrease with increasing observed midazolam plasma concentration.

**Figure 6.**
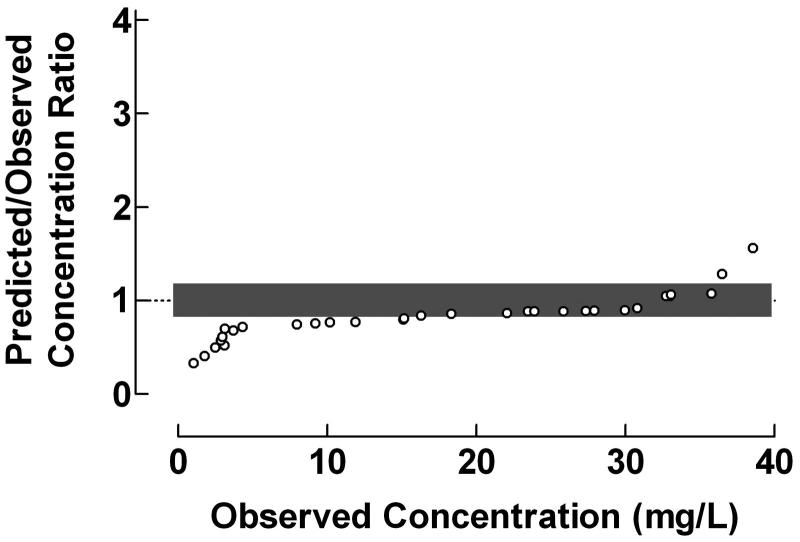
Use of the proposed ratio plot for evaluation of Bayesian forecasting performance of serum vancomycin concentrations in neonates and infants. Vancomycin was administered intravenously over 40–55 min. The population pharmacokinetics were studied using a one-compartment model. Shadowed area: Predicted/Observed concentration ratio within the range 0.8–1.2. Data from Rodvold *et al*.13.

The use of the proposed plot for evaluation of the predictive performance of Bayesian forecasting was exemplified by data from a study of serum vancomycin concentrations in neonates and infants13, Figure 6. Vancomycin was administered intravenously over 40–55 min and the population pharmacokinetics were studied using a one-compartment model. The Predicted/Observed drug concentration ratios were fairly constant within the range 3–30 mg/L, but decreased drastically with decreasing observed drug concentrations below this range and increased with increasing observed drug concentrations above it. The fraction of concentration ratios within the range 0.8–1.2 was 48.4%. The results in Figure 6 indicate that the used one-compartment model might be less suitable since the Bayesian forecasting only gives reliable estimation of vancomycin concentration within the range 15–35 mg/L.

**Figure 7.**
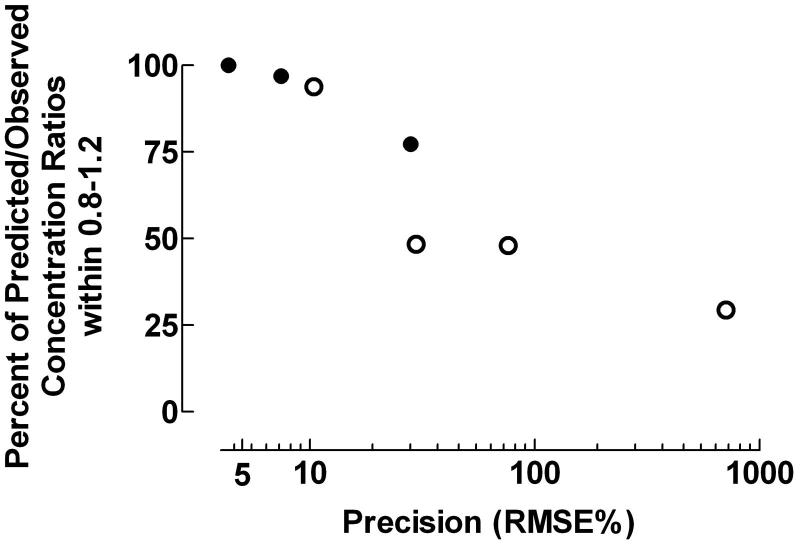
Fraction of data points within the Predicted/Observed drug concentration ratio 0.8–1.2 and precision expressed as the percentage root mean square prediction error (RMSE%). • = data from individual pharmacokinetic modeling8–10; ˆ = data from population pharmacokinetic studies11–13. *r*_s_ = −1.000, *p* = 0.0004.

The predictive performance expressed as the fraction of data points within the Predicted/Observed drug concentration ratio 0.8–1.2 decreased with increasing precision expressed as RMSE% (*r*_s_ = −1.000, *p* = 0.0004), Figure 7.

## Discussion

Pharmacokinetic studies are important for optimizing drug treatment, but proper validation of the used pharmacokinetic procedures is necessary to avoid biased and imprecise parameter estimates. The plot method proposed in the present paper is a valuable tool to illustrate the predictive performance of individual and population compartment pharmacokinetic analysis.

The precision of the pharmacokinetic processes evaluated by the fraction of data points within the Predicted/Observed drug concentration ratio 0.8–1.2 and RMSE% were closely correlated (Figure 7), but only the evaluation method proposed here revealed time- or concentration-dependent inaccuracies, as illustrated in Figures 1–6. Quantification of the predictive performance by the fraction of Predicted/Observed drug concentration ratios within specified ranges is not sensitive to outliers in contrast to evaluation of precision by RMSE% and bias by MPE%17,18. The squaring process used by Sheiner and Beal5 may also produce non-normal distribution of errors with possible bias in percentage prediction error, which might violate the assumptions of standard parametric statistical procedures17,18.

Population pharmacokinetic approaches can optimize use of limited data. These developments have enhanced the ability to conduct comprehensive pharmacokinetic studies to define population pharmacokinetics of drugs, e.g., in the pediatric population19, otherwise limited due to practical and ethical considerations20.

The accuracy and precision of data from population pharmacokinetic evaluation generally seem to be low as compared to individual pharmacokinetic modeling. It has, albeit only in a few instances, been claimed that population pharmacokinetic data must be used with precautions in clinical practice due to the imprecision of the technique21–23. Errors in recording sampling times, dosing history, as well as methodological problems in drug delivery could result in biased and imprecise parameter estimates24,25. Low predictive performance was also demonstrated by scatter plots of predicted vs observed concentrations when population pharmacokinetic models were validated using simulated data26. The importance of informative graphics for evaluation of predictive performance of pharmacokinetic model fitting was previously emphasized27.

In conclusion, the plot method proposed in the present paper is suitable for evaluation of predictive performance of both individual and population compartment pharmacokinetic analysis. It gives valuable information concerning time- and concentration-dependent inaccuracies that might occur, and might facilitate the choice of proper pharmacokinetic models.

## Transparency

### Declaration of funding

The author received no payment in preparation of this manuscript.

### Declaration of financial/other relationships

The author declared no conflicts of interest.

## Acknowledgments

Professor Hans Ehrsson is gratefully acknowledged for valuable discussions of the manuscript.
